# When healing becomes a burden: The feedback of tunisian psychiatrists

**DOI:** 10.1192/j.eurpsy.2021.857

**Published:** 2021-08-13

**Authors:** N. Faouel, B. Ben Mohamed, M. Bejar, R. Ayoub, F. Zaafrane, L. Gaha

**Affiliations:** 1 Research Laboratory, R05es10, Faculty Of Medicine Of Monastir, Tunisia, Psychiatry Department, Fattouma Bourguiba University Hospital, Monastir, Tunisia, monastir, Tunisia; 2 Psychiatry, Faculty of medicine of Monastir, Monastir, Tunisia

**Keywords:** psychiatrists, quality of life, perceived stress

## Abstract

**Introduction:**

Psychiatry is a fascinating medical specialty. Many reasons may motivate early career doctors to choose this field. However, this experience could have a different impact on their quality of life and social functioning.

**Objectives:**

we aimed to assess the impact of psychiatry as a medical career, on the psychiatrist’s quality of life, and to evaluate their feedback on their experience and how it effects their life.

**Methods:**

This is a cross-sectional descriptive study of 68 psychiatrists. An E-questionnaire has been sent via a psychiatrist’s closed groups on social media. We collected sociodemographic data, we also used the stress perceived scale, and we explored the quality of life using the SF-12 questionnaire.

**Results:**

The participants were mostly females with a mean age of 32 years (range25-65). Only 10% of psychiatrists had psychiatric history mostly depression. Concerning substance use, 15% were smokers, 17% used alcohol, 10% smoked occasionally cannabis and 23 % used different psychotropic drugs. 73% our sample were interested in psychiatry during their studies. 60% of our population considered the role of psychiatrists ambiguous among other colleagues. A very high perceived stress was noted in 90 % of our sample. Physical health status was in the average of standard deviation wether mental health status was below average among psychiatrists. The most frustrating situation mentioned was the absence of intermediate structures to receive psychotic patients.

**Conclusions:**

Tunisians psychiatrists are facing many obstacles during the practice of their job, that would transform their passion into demotivation and a desire to leave the country.

